# CIGB-258, a Potential Novel Approach to Treat Sepsis-like Hyperinflammation, Reduces Gastrointestinal Hemorrhage in Zebrafish Exposed to Carboxymethyllysine and Ethanol

**DOI:** 10.3390/ph19030510

**Published:** 2026-03-20

**Authors:** Kyung-Hyun Cho, Yunki Lee, Sang Hyuk Lee, Ashutosh Bahuguna, María del Carmen Domínguez-Horta, Gillian Martínez-Donato

**Affiliations:** 1 Raydel HDL Research Institute, Medical Innovation Complex, Daegu 41061, Republic of Korea; 2 Center for Genetic Engineering and Biotechnology, Ave 31, e/158 y 190, Playa, Havana 10600, Cuba

**Keywords:** CIGB-258 (Jusvinza^®^), carboxymethyllysine, inflammation, intestine, interleukin-6, oxidative stress, senescence

## Abstract

**Objective:** CIGB-258 is a 3 KDa altered peptide ligand recognized for its anti-inflammatory activity. Herein, the effect of CIGB-258 was assessed against carboxymethyllysine (CML) and ethanol (Et-OH)-induced sepsis-like events in zebrafish (*Danio rerio*). **Methodology:** Adult zebrafish (n = 30/group) were intraperitoneally microinjected (10 μL) with CML (final 3 mM) + Et-OH (final 50%) or CML + Et-OH containing CIGB-258 (final 1 μM) and analyzed for swimming activity, abdominal bleeding and survivability. The zebrafish were sacrificed 180 min after injection, and blood and organs were processed for biochemical and histological evaluation. **Results:** The CML + Et-OH group showed the lowest survival, compromised swimming ability, and severe abdominal bleeding 60 min post-treatment, which were substantially improved by treatment with CIGB-258. The CML + Et-OH group showed the greatest extent of oxidization and the lowest antioxidant activity in plasma, while co-treatment with CIGB-258 resulted in a remarkable improvement in oxidative extent and antioxidant status. The CML + Et-OH group showed dyslipidemia and an atherogenic lipid profile, which were substantially prevented by the CIGB-258 treatment. The livers and kidneys of the CML + Et-OH group showed the greatest extent of inflammation and senescence, which were substantially ameliorated by treatment with CIGB-258. Similarly, the CML + Et-OH group exhibited severe intestinal bleeding, which decreased 2.2-fold following treatment with CIGB-258. H&E staining and Mason-trichrome staining revealed extreme disruption to intestinal microvillus cell morphology and severe fibrosis in the intestines of the CML + Et-OH group, which effects were mitigated by the treatment with CIGB-258. **Conclusions:** The CML + Et-OH treatment resulted in acute gastrointestinal bleeding, severe oxidative stress, and hepatic and renal damage, leading to acute septic shock-like death in zebrafish. However, treatment with CIGB-258 reduced these effects through antioxidant and anti-inflammatory actions and by increasing HDL-C levels.

## 1. Introduction

A widespread hyperinflammation and host immune response to external stimuli is associated with tissue damage and organ failure, leading to the life-threatening condition sepsis [1]. Septic shocks are the primary cause of sudden death, frequently associated with disruption of major organs, such as the lungs, liver, kidneys, gastrointestinal tract, and blood coagulation network [2,3]. Septic shock can result from infections caused by viruses, protozoa, fungi, and bacteria, which trigger the activation of immune cells to secrete proinflammatory cytokines as part of the host’s immune response to pathogen-associated factors [4,5]. It is well known that lipopolysaccharide (LPS) and advanced glycation end products (AGEs), such as carboxymethyllysine (CML), play a critical role in immune system activation by binding to toll-like receptor (TLR)-4 and the receptor for advanced glycation end products (RAGE), respectively [6,7]. This recognition triggers immune cell activation, leading to the production of various cytokines and chemokines [8]. In sepsis, cytokines play a key role in dysregulated immune responses and thus serve as important biomarkers in sepsis management [1]. Several cytokines, including interleukin (IL)-6 and tumor necrosis factor (TNF)-α, play a pivotal role in sepsis [1]. IL-6 and TNF-α are pro-inflammatory cytokines produced by activated monocytes and macrophages. They promote lymphocyte differentiation [9] and play an important role in the immune response during sepsis [1]. Despite the substantial advances in medical sciences, hyperinflammation and sepsis continue to pose a major global health challenge, contributing to high morbidity and mortality [1,10]. The experimental model of CML treatment in zebrafish reproduces several key features of sepsis, including systemic hyperinflammation, oxidative stress, and liver and kidney damage [11,12]. These manifestations are comparable to those observed in classical mammalian models of sepsis, thereby validating the zebrafish as a suitable organism for the investigation of pathogenic mechanisms and evaluation of potential therapeutic strategies against sepsis [13,14].

CIGB-258 (Jusvinza^®^) is an altered peptide ligand (APL) composed of 27 amino acids, originating from human heat shock protein (HSP) 60 [15]. In CIGB-258, amino acid no. 18 (aspartic acid) of the native peptide was substituted with leucine [15]. This point mutation imparts to CIGB-258 a high affinity towards the major histocompatibility complex type II (MHC-II) and consequently confers a notable immunomodulatory effect [15]. CIGB-258 has been well recognized for its anti-inflammatory activity and its inhibitory effect on the production of cytokines, including IL-6 and TNF-α [16,17,18]. Clinical outcomes in rheumatoid arthritis (RA) indicate that CIGB-258 effectively counteracts inflammation by inducing the proliferation of immunosuppressive regulatory T cells (T_reg_), inhibiting proinflammatory IL-17, and restoring the normal Th17/T_reg_ balance [15]. Notably, CIGB-258 demonstrated substantial potential to treat COVID-19 by suppressing hyperinflammation [16]. It reduces the efflux of proinflammatory cytokines IL-6 and TNF-α, as well as granzyme and perforin, while simultaneously upregulating T_reg_ cells in COVID-19 patients [16]. Beyond its direct immunoregulatory effects, CIGB-258 also positively impacts HDL stability [11,19,20] and functionality [11,20]. This represents an additional mechanism of immunomodulation, as HDL regulates both innate and adaptive immune responses and has a therapeutic relevance in inflammatory diseases [21].

Despite the promising role of CIGB-258 in inflammatory disorders, its effects on sepsis-like conditions have not been assessed. Concerning this, the present study was designed to examine the effect of CIGB-258 on acute toxicity (marked by impairment of swimming activity, survivability, and gastrointestinal bleeding and damage to the liver, kidneys and intestine) triggered by intraperitoneal exposure to CML and ethanol (CML + Et-OH) using zebrafish. In addition, the effect of CIGB-258 on the plasma lipoprotein profiles and oxidative variables of zebrafish exposed to a sepsis-like condition by treatment with CML + Et-OH was examined.

The zebrafish was selected as a model organism due to its high genetic and physiological resemblance to humans [22]. Also, many cell types, tissues and pathways involved in human diseases are similar to those involved in diseases in zebrafish [23]. In addition, zebrafish share many similarities with mice in terms of immune responses, cytokine production, and organ function [8]. However, unlike most rodents, the smaller size of zebrafish (~3–5 cm) [24] and their easy husbandry allow the management of many fishes in a small cage. Due to the aforementioned qualities, zebrafish are gaining acceptance as efficient model organisms for inflammation [22,23] and sepsis [8,25]. Therefore, the outcomes from zebrafish studies can provide invaluable information that may help to shape human studies.

## 2. Results

### 2.1. Treatment with CIGB-258 Prevents Acute Inflammatory Death

**Figure 1:**
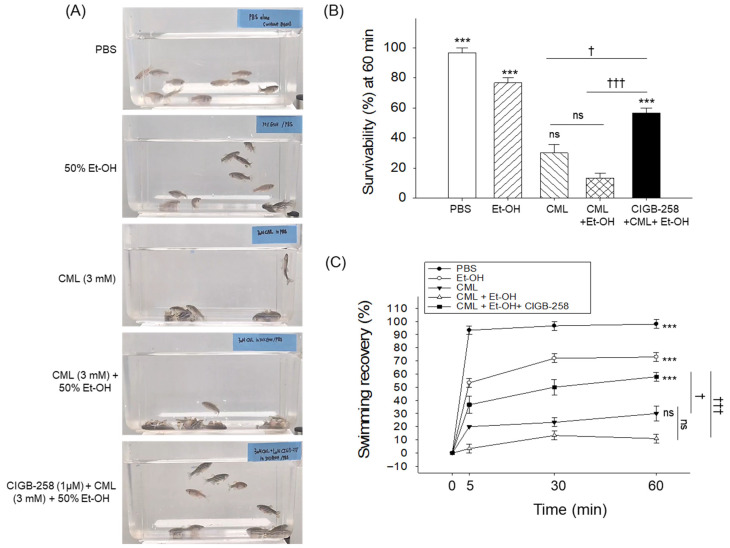
Survivability and swimming ability of zebrafish following the intraperitoneal injection of ethanol (Et-OH) and carboxymethyllysine (CML), individually and in combination with CIGB-258. (**A**) Snapshots of the swimming patterns at 60 min post-injection. Text shown in the blue boxes is positioned on the left side of panel (**A**). (**B**) Survivability and (**C**) kinetics of swimming recovery 60 min post-injection. Each data point in the line and bar graphs represents the mean ± SEM (n = 30). *** (*p* < 0.001) indicates a significant difference compared to CML + Et-OH determined via one-way ANOVA followed by Tukey’s post hoc analysis. ^†^ (*p* < 0.05) and ^†††^ (*p* < 0.001) indicate significant pairwise differences between the marked groups determined via a *t*-test; “ns” indicates a non-significant difference.

The intraperitoneal injection of CML (final 3 mM) + Et-OH (final 50%) led to severe loss of swimming ability (Figure 1A) and the lowest survival, around 13% at 60 min post-treatment (Figure 1B), whereas the PBS (control) group showed 97% survivability. In contrast to the combined treatment (CML + Et-OH), the individual treatments with Et-OH and CML alone showed 77% and 30% survival at 60 min post-injection, indicating that the combination of CML + Et-OH led to greater inflammatory death. Notably, the CIGB-258-injected group showed around 57% survivability, which is significantly (4.2-fold) higher than the survivability observed in the CML + Et-OH group, suggesting the protective effect of CIGB-258 against CML + Et-OH-induced acute death in zebrafish.

The swimming activity of zebrafish varied substantially across the groups (Figure 1A,C and Supplementary Videos S1–S5). As depicted in Figure 1C and the Supplementary Videos, the PBS-injected zebrafish displayed 93% swimming recovery 5 min post-treatment, which slightly increased to 98% at 60 min post-injection. The Et-OH-injected group displayed 53.3% swimming recovery after 5 min post-treatment, which increased to 72% at 30 min and thereafter remained constant. In contrast, only 30% swimming recovery was observed in the CML-treated group 60 min post-injection. The combined treatment of CML + Et-OH resulted in severely compromised swimming recovery, with only 11% at 60 min post-treatment, accounting for 6.6-fold and 2.7-fold lower swimming recovery in the Et-OH and CML-injected groups, respectively. Treatment with CIGB-258 significantly improved the swimming ability compromised by CML + Et-OH, as demonstrated by 3.8-fold and 5.2-fold increases in swimming ability at 30 min and 60 min post-treatment, respectively.

Notably, treatment with CIGB-258 alone did not produce any adverse effects on the swimming ability or mortality of the zebrafish. The effect of the CIGB-258-alone treatment on swimming performance and survival was statistically comparable to that observed in the PBS control group (Supplementary Figure S1 and Supplementary Video S6). 

No significant differences in body weight were detected between baseline (body weight before treatment) and body weight at 30–180 min post-treatment across all groups. Additionally, no signs of aggression were observed among zebrafish in any of the experimental groups.

### 2.2. Treatment with CIGB-258 Reduced Abdominal Bleeding

**Figure 2:**
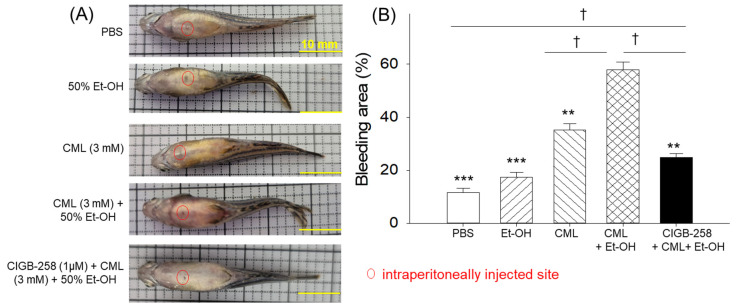
Intraperitoneal injection sites and bleeding areas of the zebrafish from different groups that received the specified treatments. (**A**) Representative images of the zebrafish abdominal injection sites and (**B**) quantification of the abdominal bleeding areas 180 min after treatment. Abbreviations: PBS: phosphate-buffered saline; Et-OH: ethanol; CML: carboxymethyllysine; CIGB-258: peptide. Data points in the bar graph represent the mean ± SEM (n = 30). ** (*p* < 0.01) and *** (*p* < 0.001) highlight the statistical differences to the CML + Et-OH group determined via one-way ANOVA following Tukey’s post hoc analysis; ^†^ (*p* < 0.05) highlights the statistical difference between the marked groups determined via a *t*-test.

As shown in Figure 2, the PBS-injected (control) group showed the least blood accumulation in the abdomen area. In contrast, the individual treatments with Et-OH and CML showed increased red intensity in the abdomen area, this being 1.5-fold and 3.0-fold higher, respectively, than that in the PBS group. In contrast to the individual treatments, the combined treatment with CML + Et-OH resulted in severe bleeding in the abdominal area. The bleeding area was approximately 3.3-fold and 1.6-fold higher than that observed in the Et-OH and CML groups, respectively. These results indicate an enhanced effect of the CML + Et-OH combination in causing severe inflammation. Treatment with CIGB-258 effectively protected against CML + Et-OH-induced inflammation, resulting in a 2.3-fold decrease in bleeding area. Zebrafish treated with CIGB-258 alone showed abdominal bleeding comparable to the PBS (control) group, indicating no adverse effect of CIGB-258 on the induction of abdominal bleeding (Supplementary Figure S2).

### 2.3. Comparison of Oxidization Extent and Antioxidant Activities in Zebrafish Plasma

**Figure 3:**
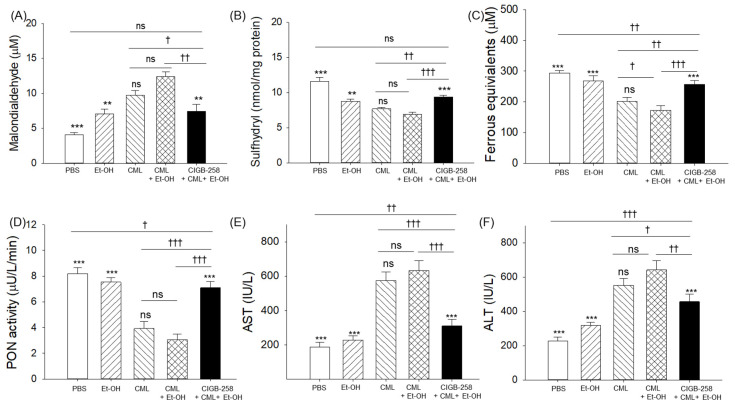
A comparative study of oxidative variables, antioxidant parameters, and liver function biomarkers in plasma from the zebrafish that received the specified treatment. Plasma (**A**) malondialdehyde, (**B**) sulfhydryl, (**C**) ferrous equivalent, (**D**) paraoxonase (PON), (**E**) aspartate aminotransferase (AST), and (**F**) alanine aminotransferase (ALT) levels 180 min post-treatment. Abbreviations: PBS: phosphate-buffered saline; Et-OH: ethanol; CML: carboxymethyllysine; CIGB-258: peptide. Data points in the bar graph represent the mean ± SEM (n = 3). ** (*p* < 0.01) and *** (*p* < 0.001) highlight the statistical differences to CML + Et-OH determined via one-way ANOVA following Tukey’s post hoc analysis; ^†^ (*p* < 0.05), ^††^ (*p* < 0.01) and ^†††^ (*p* < 0.001) highlight the statistical differences between the marked groups determined via a *t*-test; “ns” represents a non-significant difference.

As shown in Figure 3A, quantification of oxidized species in the zebrafish plasma using thiobarbituric acid reactive substances (TBARS) revealed that the CML + Et-OH group showed the highest content of malondialdehyde (MDA), which was significantly (3.1-fold) higher than the basal MDA level in the PBS (control) group. Treatment with CIGB-258 with CML + Et-OH resulted in a 1.6-fold decrease in MDA levels relative to the treatment with CML + Et-OH, suggesting the protective effect of CIGB-258 against CML + Et-OH-induced oxidative stress.

Quantification of the sulfhydryl content in the plasma using Ellman’s reagent revealed that the CML + Et-OH group showed the lowest sulfhydryl content (6.8 ± 0.3 nmol/mg), around 1.7-fold lower than the basal level detected in the PBS (control) group (11.6 ± 0.6 nmol/mg) (Figure 3B). Treatment with CIGB-258 with CML + Et-OH resulted in a 1.4-fold increase in sulfhydryl content, suggesting that CIGB-258 has a potent reduction ability in the presence of CML + Et-OH.

Ferric ion reduction ability (FRA) was highest (293.6 ± 8.2 μM) in the PBS (control) group and lowest in the CML + Et-OH group (172.4 ± 14.7 μM), the value being significantly (1.7-fold) lower than that in the PBS group (Figure 3C). The treatment with CIGB-258 substantially elevated (257.2 ± 12.6 μM) the CML + Et-OH-diminished FRA activity. Compared to the CML + Et-OH group, the CIGB-258-injected group displayed 1.5-fold higher FRA activity that was statistically similar to the FRA activity observed in the PBS (control) group.

As shown in Figure 3D, the individual treatments with Et-OH and CML resulted in 1.1-fold and 2.1-fold lower paraoxonase (PON) activity than the PBS (control) group. In contrast, the combination of CML + Et-OH exhibited a more severe effect in terms of diminishing PON activity, which was significantly (2.6-fold) lower than the basal PON activity observed in the PBS (control) group. The CIGB-258 treatment resulted in a 2.3-fold enhancement in the CML + Et-OH-diminished PON activity.

Importantly, only in the CIGB-258-treated groups did plasma levels of MDA, sulfhydryl contents, and FRA and PON activities show values statistically similar to those observed in the PBS-treated groups, highlighting no adverse effect of CIGB-258 on the plasma oxidative and antioxidant variables (Supplementary Figure S3).

The combined results suggest exacerbated oxidization extent and decreased antioxidant abilities in response to CML + Et-OH, which effects were substantially improved by the treatment with CIGB-258. The highest antioxidant abilities and the least extent of oxidative stress in response to CIGB-258 could have contributed to the highest swimming recovery (Figure 1C) and the lowest abdominal bleeding (Figure 2).

### 2.4. Hepatic Enzyme Levels in Plasma

The CML + Et-OH group showed the highest AST (632.2 ± 58.9 IU/L) and ALT (641.8 ± 53.4 IU/L) levels, which were significantly (3.3-fold and 2.8-fold) higher than their respective levels observed in the PBS (control) group. These findings indicate a substantial hepatic-damaging effect of CML + Et-OH exposure (Figure 3E,F). Treatment with CIGB-258 resulted in a significant 2.1-fold and 1.4-fold reduction in the CML + Et-OH-elevated AST and ALT levels, respectively. The results suggest the hepatoprotective role of CIGB-258 towards CML + Et-OH-posed toxicity. Notably, exposure to CIGB-258 alone did not produce any adverse effect on plasma AST or ALT levels. These levels were statistically similar to the AST and ALT levels observed in the PBS (control) group (Supplementary Figure S3).

### 2.5. Change in Lipid Profiles in Plasma

**Figure 4:**
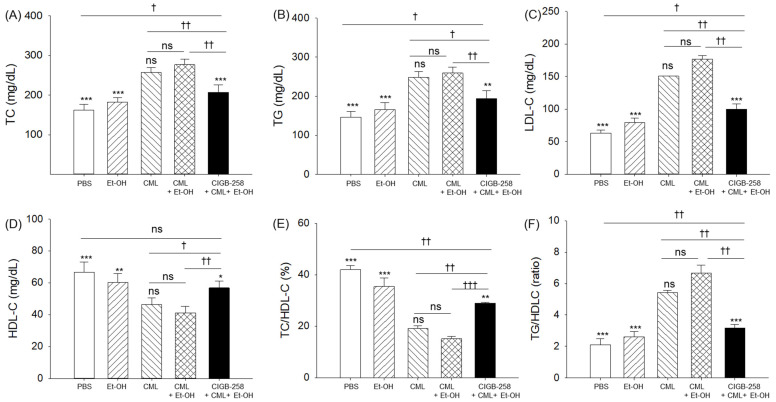
A comparison of plasma lipoprotein profiles of zebrafish that received specified treatments. Plasma levels of (**A**) total cholesterol (TC), (**B**) triglycerides (TGs), (**C**) low-density lipoprotein cholesterol (LDL-C), and (**D**) high-density lipoprotein cholesterol (HDL-C), as well as (**E**) percentage ratio of TC/HDL-C and (**F**) ratio of TG/HDL-C at 180 min post-treatment. Abbreviations: PBS: phosphate-buffered saline; Et-OH: ethanol; CML: carboxymethyllysine; CIGB-258: peptide. Data points in the bar graph represent the mean ± SEM (n = 3). * (*p* < 0.05), ** (*p* < 0.01) and *** (*p* < 0.001) highlight the statistical differences to the CML + Et-OH groups determined via one-way ANOVA following Tukey’s post hoc analysis; ^†^ (*p* < 0.05), ^††^ (*p* < 0.01) and ^†††^ (*p* < 0.001) highlight the statistical differences between the marked groups determined via a *t*-test; “ns” represents a non-significant difference.

As shown in Figure 4, the CML + Et-OH group showed the highest plasma total cholesterol (TC, 276.9 ± 13.4 mg/dL) and triglyceride (TGs, 259.5 ± 14.8 mg/dL) levels, which were ~1.7-fold higher than the respective levels observed in the PBS (control) group. In contrast to the combined CML treatment, exposure to Et-OH alone did not significantly increase TC or TG levels. However, individual exposure to CML substantially elevated TC and TG levels, which were 1.5-fold and 1.6-fold higher than those observed in the PBS (control) group, highlighting CML’s strong impact on TC and TG elevation. The CIGB-258 exposure showed a ~1.3-fold decrease in the CML + Et-OH-elevated TC and TG levels, respectively, indicating a positive effect of CIGB-258 in terms of downregulating the lipid profile from the proinflammatory response induced by CML + Et-OH.

In addition to the elevation of TC and TGs, the CML + Et-OH group showed the highest level of LDL-C (177.1 ± 4.9 mg/dL), which was significantly (2.8-fold) higher than the LDL-C level in the PBS (control) group (Figure 4C). The CML + Et-OH-induced elevated LDL-C level was substantially countered by CIGB-258 exposure, as evidenced by a 1.8-fold lower LDL-C level in the CIGB-258-treated group compared with the CML + Et-OH group.

In contrast to the highest LDL-C level, the lowest plasma HDL-C level (41.1 ± 4.1 mg/dL) was detected in the CML + Et-OH-injected groups, this result being significantly (1.6-fold) lower than the HDL-C level detected in the PBS control group (66.7 ± 6.4 mg/dL) (Figure 4D). Compared with the individual effects of Et-OH and CML, their combined exposure showed 1.5-fold and 1.1-fold lower HDL-C levels, respectively, indicating that the combination CML + Et-OH leads to a greater decline in HDL-C. The CIGB-258-injected group showed a notable 1.4-fold elevation in HDL-C levels, which was diminished by exposure to CML + Et-OH. Similarly, the lowest TC/HDL-C level (%) was observed in the CML + Et-OH group, which was significantly elevated by CIGB-258 treatment (Figure 4E). Moreover, the CML + Et-OH-treated group exhibited the highest TG/HDL-C ratio, around 3.2-fold higher than the PBS (control) group (Figure 4F), which was subsequently decreased 2.1-fold by the co-treatment with CIGB-258.

The findings suggest that CIGB-258 effectively mitigates the CML + Et-OH-induced elevation of TC, TGs, and LDL-C, while restoring HDL-C levels. These results attest to the potential of CIGB-258 to maintain the plasma lipoprotein profile disrupted by acute exposure to CML + Et-OH.

### 2.6. Histological Analysis of Hepatic Sections

**Figure 5:**
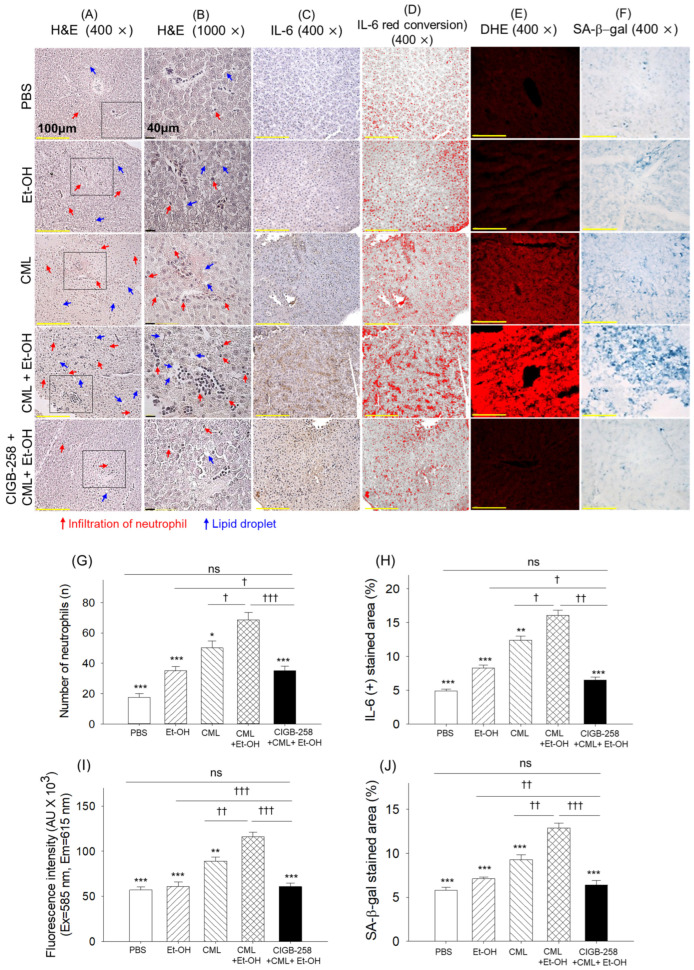
Histological examination of zebrafish following the specified treatments after 180 min. (**A**) Hematoxylin and eosin (H&E) staining at 400× magnification. (**B**) Magnified views (1000×) of the H&E-stained areas within the black boxes. A semiquantitative assessment of dark violet-stained neutrophils was performed by microscopically examining the designated area (1.23 mm^2^) across three sections and five fields in each group. (**C**) Interleukin (IL)-6 detection by immunocytochemistry (IHC). (**D**) Red conversion of the IL-6-stained areas using ImageJ software (https://imagej.net/ij, version 1.53; accessed on 6 June 2025) at a brown color threshold value (20–120) was performed to enhance the intensity of the stained area. (**E**,**F**) Dihydroethidium (DHE) fluorescent staining and senescent-associated β-galactosidase (SA-β-gal) staining, respectively. Quantification of (**G**) neutrophils, (**H**) IL-6 stained area, (**I**) DHE fluorescent intensity, and (**J**) SA-β-gal stained area. Abbreviations: PBS: phosphate-buffered saline; Et-OH: ethanol; CML: carboxymethyllysine; CIGB-258: peptide. Values in the bar graphs represent the mean ± SEM from three different sections (n = 3). * (*p* < 0.05), ** (*p* < 0.01) and *** (*p* < 0.001) highlight the statistical differences to the CML + Et-OH groups determined via one-way ANOVA following Tukey’s post hoc analysis; ^†^ (*p* < 0.05), ^††^ (*p* < 0.01) and ^†††^ (*p* < 0.001) highlight the statistical differences between the marked groups determined via a *t*-test; “ns” represents a non-significant difference.

A massive neutrophil infiltration, which was significantly (3.8-fold) higher than the basal neutrophil counts of the PBS (control) group, was observed in the CML + Et-OH-injected zebrafish (Figure 5A,B,G). Individual treatments with Et-OH and CML also increased hepatic neutrophil counts; however, compared with their combined treatment (CML + Et-OH), the individual effects were significantly inferior. In contrast, the CIGB-258 treatment effectively prevented the CML + Et-OH-induced neutrophil efflux, as evidenced by a significantly (2-fold) reduced neutrophil count in the CIGB-258-treated group compared to the CML + Et-OH group.

Consistent with the neutrophil outcomes, heightened IL-6 production was observed in the CML + Et-OH groups, which was significantly (3.2-fold) higher than the basal IL-6 level (PBS group) and 1.9-fold and 1.2-fold higher than the levels in the groups injected individually with Et-OH and CML (Figure 5C,D,H). The co-exposure to CML + Et-OH with CIGB-258 significantly minimized IL-6 production by 2.5-fold relative to the CML + Et-OH-injected group.

The DHE and SA-β-gal staining revealed a ~2.1-fold increase in ROS generation and cellular senescence in the CML + Et-OH-injected group compared to their respective basal levels in the PBS (control) group (Figure 5E,F,I,J). The treatment with CIGB-258 substantially inhibited the CML + Et-OH-induced ROS generation and senescence by 1.9-fold and 2-fold, respectively.

Notably, neutrophil counts, IL-6 production, ROS generation, and senescence in the CIGB-258-injected group were statistically similar to those in the PBS (control) group, attesting to CIGB-258’s high efficacy in restoring the CML + Et-OH-triggered events to basal levels. 

### 2.7. Histological Analysis of Kidneys

**Figure 6:**
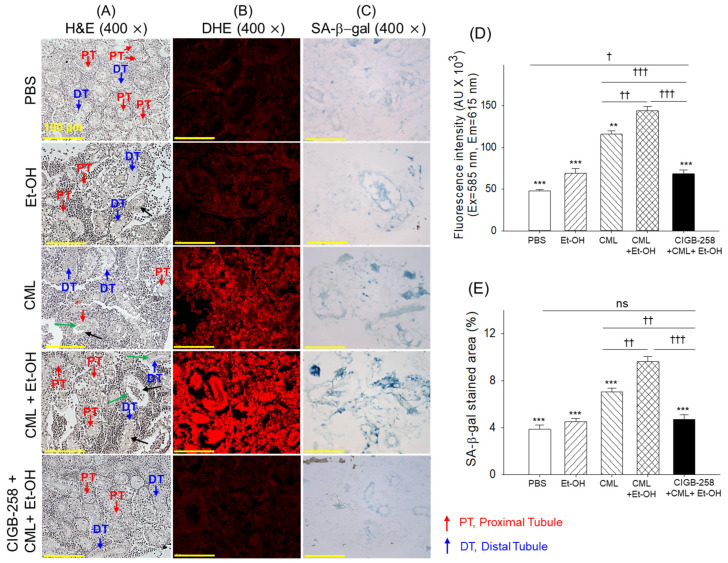
Histological analysis of the kidneys from the zebrafish that received specified treatments after 180 min. (**A**) Hematoxylin and eosin (H&E) staining at 400× magnification. (**B**) Dihydroethidium (DHE) fluorescent staining and (**C**) and senescent-associated β-galactosidase (SA-β-gal) staining. Quantification of (**D**) DHE fluorescent intensity and (**E**) SA-β-gal-stained area. Abbreviations: PBS: phosphate-buffered saline; Et-OH: ethanol; CML: carboxymethyllysine; CIGB-258: peptide. Values in the bar graph represent the mean ± SEM from three different sections (n = 3). ** (*p* < 0.01) and *** (*p* < 0.001) highlight the statistical differences to the CML + Et-OH groups determined via one-way ANOVA following Tukey’s post hoc analysis; ^†^ (*p* < 0.05), ^††^ (*p* < 0.01) and ^†††^ (*p* < 0.001) highlight the statistical differences between the marked groups determined via a *t*-test; “ns” highlights a non-significant difference.

As shown in Figure 6A, H&E staining revealed highly dense and organized proximal tubules (PTs, highlighted by red arrows) and distal tubules (DTs, highlighted by blue arrows) in the kidney sections of the PBS-injected group. In contrast to this, substantial changes in tubular structure and arrangement appeared in the Et-OH- and CML-injected groups. However, the combined supplementation with CML + Et-OH displayed a more severe effect, as reflected by the highly distorted and sparsely populated tubular structures with broad tubular lumens (indicated by green arrows) and cellular debris in the tubular lumens (indicated by black arrows). The treatment with CIGB-258 effectively inhibited the CML + Et-OH-induced histological changes in the kidneys and preserved kidney cellular integrity; however, rare cellular debris and dilated tubular lumens were also observed.

The DHE and SA-β-gal staining showed the highest DHE fluorescence intensity, and the blue-stained cells correspond to ROS generation and cellular senescence in the kidneys of the CML + Et-OH-injected group, which were notably 3-fold and 2.5-fold higher than their corresponding basal levels in the PBS (control) group (Figure 6B–E). Overall, treatment with CIGB-258 protects the kidneys from CML + Et-OH-induced ROS generation and cellular senescence.

### 2.8. Intestinal Bleeding and Congestion

**Figure 7:**
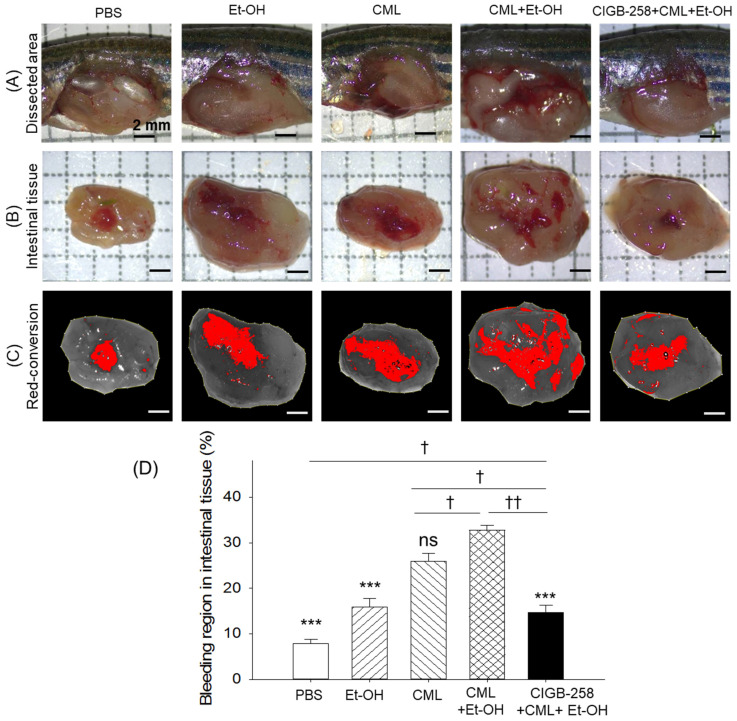
Comparison of bleeding and swollen areas in the zebrafish intestinal tissue at the intraperitoneal site of the abdomen 180 min post-treatment with the specified treatments. (**A**) Dissected areas of the lower abdominal regions. (**B**) Stereo images of the intestinal tissue near the intraperitoneal sites, highlighting bleeding and swollen areas. (**C**) Red conversion of bleeding areas using Image J software (https://imagej.net/ij, version 1.53; accessed on 6 June 2025) at a red color threshold value (20–120). (**D**) Quantification of the bleeding regions in the intestinal tissue. The red conversion was performed to enhance the visibility of bleeding sites. Abbreviations: PBS: phosphate-buffered saline; Et-OH: ethanol; CML: carboxymethyllysine; CIGB-258: peptide. Values in the bar graph represent the mean ± SEM from three different sections (n = 3). *** (*p* < 0.001) highlights the statistical differences to the CML + Et-OH groups determined via one-way ANOVA following Tukey’s post hoc analysis; ^†^ (*p* < 0.05) and ^††^ (*p* < 0.01) highlight the statistical differences between the marked groups determined via a *t*-test.

As depicted in Figure 7A–D, the least amount of intestinal bleeding was observed and quantified in the PBS-injected group, which was significantly (4.2-fold) lower than the bleeding detected in the CML + Et-OH group. Individually, Et-OH treatment does not have any substantial effect on intestinal bleeding, but the insertion of CML substantially enhances intestinal bleeding. CML in combination with Et-OH (CML + Et-OH) leads to severe intestinal bleeding that is significantly (2.1-fold and 1.2-fold) higher than the bleeding effect exerted by treatment with Et-OH and CML, respectively. The CML + Et-OH-triggered intestinal bleeding was markedly attenuated by the treatment with CIGB-258, as indicated by a 2.2-fold reduced bleeding area in the CIGB-258-injected group relative to the CML + Et-OH group.

### 2.9. Intestinal Histology

**Figure 8:**
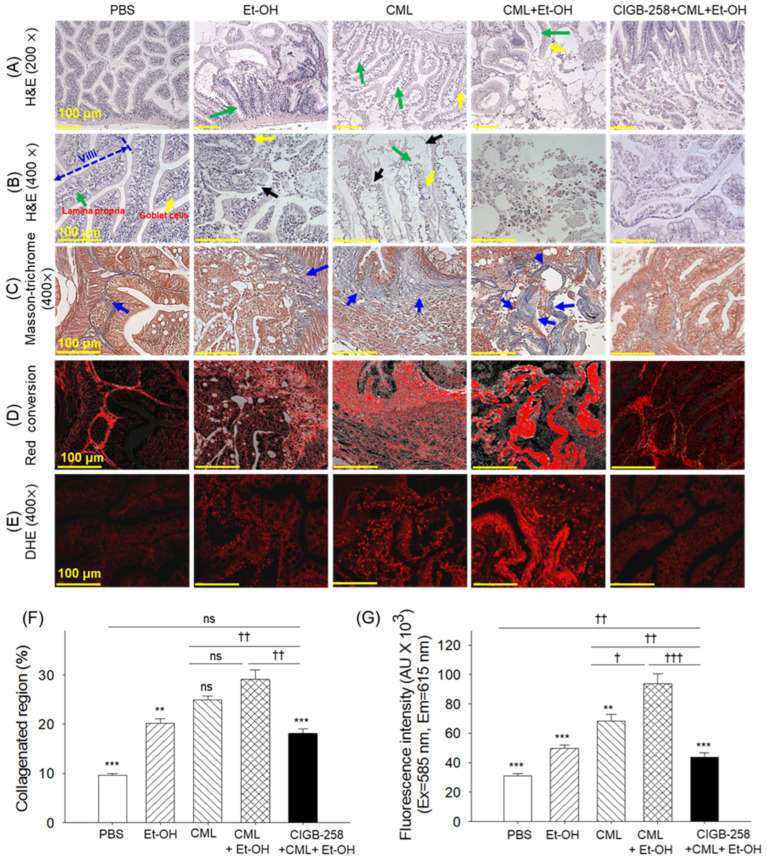
Histological analysis of intestinal tissue from the zebrafish that received specified treatments after 180 min. (**A**,**B**) Hematoxylin and eosin (H&E)-stained sections at 200× and 400× magnifications, respectively. Green, black, and yellow arrows highlight degeneration of the lamina propria, enteric villus dissolution, and swelling or shrinkage of goblet cells, respectively. (**C**) Mason-trichrome staining; blue arrows highlight the collagenated regions. (**D**) Red conversion of the blue-stained collagen that appeared in the Mason-trichrome staining. To enhance visibility, red conversion was performed using ImageJ software (https://imagej.net/ij, version 1.53; accessed on 6 June 2025) with a blue color threshold of 20–120. (**E**) Dihydroethidium (DHE) fluorescent staining. (**F**,**G**) Quantification of the collagenated regions that appeared in the Masson-trichrome-stained sections and DHE fluorescent intensity, respectively. Abbreviations: PBS: phosphate-buffered saline; Et-OH: ethanol; CML: carboxymethyllysine; CIGB-258: peptide. Values in the bar graphs represent the mean ± SEM from three different sections (n = 3). ** (*p* < 0.01) and *** (*p* < 0.001) highlight the statistical differences to the CML + Et-OH groups determined via one-way ANOVA following Tukey’s post hoc analysis; ^†^ (*p* < 0.05), ^††^ (*p* < 0.01) and ^†††^ (*p* < 0.001) highlight the statistical differences between the marked groups determined via a *t*-test; “ns” highlights a non-significant difference.

The intestinal H&E staining of the PBS-injected group revealed a properly arranged villus structure with an intact lamina propria (Figure 8A,B). The individual treatments with Et-OH and CML showed morphological changes, including dissolution of enteric villi (indicated by black arrows) and swelling or shrinkage of goblet cells (indicated by yellow arrows). Compared to the individual treatments, the combined treatment of CML + Et-OH resulted in severe disruption to villus integrity, degeneration of the lamina propria (indicated by green arrows), and hypertrophy of goblet cells. Treatment with CIGB-258 substantially protected against acute intestinal damage; however, the occasional presence of disrupted villi and hypertrophic goblet cells was also observed, though these changes were substantially inferior to those in the CML + Et-OH group.

The Mason-trichrome staining revealed a low-collagenated region in the PBS-injected group, which was substantially elevated by 2.1-fold and 2.5-fold after treatment with Et-OH and CML, respectively, relative to the PBS group (Figure 8C,D,F). However, the combined treatment of CML + Et-OH substantially increased the collagenated regions (indicated by blue arrows); the levels of collagenation were 3-fold higher than the basal level observed in the PBS-injected group, attesting to the disruption of the inner circular muscle and the accumulation of fibrous connective tissue. In response to the CML + Et-OH-triggered injury, the CIGB-258 treatment demonstrated the protection of circular muscle tissue fibrosis, highlighted by a 1.6-fold-reduced collagenated region compared to the CML + Et-OH-injected group.

ROS production (DHE fluorescence) was 3.1-fold higher in the CML + Et-OH group compared to the basal levels detected in the PBS (control) group. Co-treatment with CIGB-258 markedly reduced elevated levels of ROS, as evidenced by the 2.2-fold-reduced DHE fluorescence compared to the CML + Et-OH group.

## 3. Discussion

Hyperinflammation has been recognized to cause severe adverse impacts on tissue and organs [26], leading to sepsis-like conditions [27]. In sepsis, inflammation is induced by a variety of cytokines and oxygen radicals [28]. A high efflux of neutrophils, which release ROS and a variety of proteases, amplifies the inflammatory response in sepsis [28]. Accumulation of AGEs, such as CML, stimulates NADPH oxidases and activates NF-κB signaling, thereby prompting the inflammatory response [29]. Besides provoking inflammation, CML effects ROS generation, and oxidative stress has been recognized [30]. Several clinical studies have demonstrated that higher levels of AGEs are associated with sepsis severity. In the context of septic shock, CML is widely recognized as a key external factor in triggering the inflammatory cytokine storm in chronic kidney disease and type 2 diabetes mellitus [30]. However, higher intake of AGEs and ethanol is generally considered to exacerbate sepsis by altering the activation of monocytes, macrophages, and dendritic cells [28,31]. Ethanol exposure especially dysregulates the innate immune response, leading to increased morbidity and mortality in patients with sepsis [32]. Herein, a severe impact of the CML + Et-OH treatment was noticed on the acute mortality and swimming impairment of the zebrafish. It is important to note that ethanol does not merely act as a vehicle in this model. Et-OH alone induces intestinal damage and oxidative stress, and when combined with CML, it amplifies the inflammatory cascade, leading to higher IL-6 levels, neutrophil infiltration, and ROS generation. This combined effect highlights the role of Et-OH as a cofactor that exacerbates pathology, thereby strengthening the translational relevance of the zebrafish CML + Et-OH model as a sepsis-like condition.

The treatment with CIGB-258, however, demonstrated great resilience against CML-caused adverse events. These findings suggest that the anti-inflammatory properties of CIGB-258 drive cellular events that protect zebrafish from CML-induced swimming impairment and acute death. This notion is supported by reports documenting an inverse correlation between inflammation, paralysis, and acute mortality [20,33]. Also, the study revealed that inhibition of pro-inflammatory cytokines like TNF-α (using infliximab and etanercept) and IL-6 (using tocilizumab) showed great promise in improving the CML-induced severe mortality and swimming abnormalities in zebrafish, strengthening the notion that CML-induced inflammation is a major cause of acute death [20]. Nevertheless, detailed mechanistic molecular investigations are required to elucidate the precise pathways through which CIGB-258 confers protection against CML-induced acute death and impaired swimming behavior.

Besides an acute inflammatory effect, heightened oxidative stress, as measured by MDA and sulfhydryl levels, and compromised antioxidant status, concerning the PON and FRA activities, were observed in the CML + Et-OH-injected zebrafish. The outcomes are in good agreement with previous reports depicting the impact of CML on oxidative stress and impairment of the antioxidant system [29]. MDA is a key lipid peroxidation product, and its high levels are widely recognized as indicators of oxidative damage [34]. In contrast, FRA reflects the total antioxidant capacity of blood, with higher levels indicating superior antioxidant capacity [35]. The sulfhydryl groups act as primary antioxidants, neutralizing peroxyl radicals [36,37], and diminished levels have been associated with inflammatory conditions and renal disorders [38,39]. PON, an HDL-linked enzyme, plays a protective role against lipid oxidation [40], and its decreased level is associated with myocardial infarction [41] and liver disease [42]. Herein, CML + Et-OH-induced oxidative stress and compromised antioxidant defenses are prevented by exposure to CIGB-258, as reflected by decreased MDA levels and elevated sulfhydryl contents, FRA, and PON activities. Previous studies have reported that CIGB-258 inhibits lipoprotein oxidation [43] and protects zebrafish embryos from oxidative stress and apoptosis [20]. These findings highlight CIGB-258’s cellular antioxidant properties and further support the results of the present study.

The inflammatory and oxidative stress-promoting nature of CML is among the key reasons for dyslipidemia, as several reports describe an association between inflammation and the development and progression of metabolic disease [44] and dyslipidemia [45]. Consistently, we noticed severe dyslipidemia in response to CML + Et-OH exposure, marked by elevated levels of TC, TGs, and LDL-C and diminished HDL-C levels. Exposure to CIGB-258 significantly reversed the CML-induced dyslipidemia and substantially elevated HDL-C levels. As CIGB-258 has a strong anti-inflammatory effect [15,17], it prevented CML-induced dyslipidemia in zebrafish. This notion is consistent with earlier reports showing a positive correlation between elevated TG levels and the proinflammatory cytokine IL-6 [46,47]. Also, the significant impact of inflammation on HDL-C levels and HDL functionality has been recognized [45]. Precisely, elevated levels of proinflammatory cytokines like TNF-α and IL-6 are inversely correlated with serum HDL-C levels [48,49] and HDL-associated antioxidant PON activity [45]. The studies aligned with previous reports suggest a positive effect of CIGB-258 in terms of protecting the structural stability of HDL and maintaining blood cholesterol and triglyceride levels disturbed by external stress [20,43]. Studies have demonstrated an important association between lipoproteins, specifically HDL, and sepsis [50]. Sepsis profoundly disrupts HDL metabolism; consequently, a marked acute drop in HDL-C levels is observed [51,52,53]. Decreased HDL-C levels are closely associated with multi-organ failure, prolonged hospitalization and increased mortality [50]. Experimental evidence from a cecal ligation and puncture (CLP)-induced sepsis model shows that reduced HDL levels correlated with higher mortality [50,54]. Furthermore, evidence from preclinical and clinical studies suggests that external HDL administration is a potential therapeutic approach for the management of sepsis [50]. In the present study, treatment with CIGB-258 significantly increased HDL-C levels, suggesting a protective effect through restoration of HDL-C that attenuates CML + Et-OH-induced sepsis-like pathology in zebrafish.

CML is known to induce hepatic inflammation, and inflammatory markers lead to liver damage [55]. Consistently, fatty liver change and severe inflammation, evident from massive neutrophil infiltration and high IL-6 production, were observed in the CML + Et-OH-injected group and were prevented by treatment with CIGB-258. In addition, CML + Et-OH elevated AST and ALT levels, well-known hepatic function biomarkers [56], which are substantially reduced by CIGB-258 treatment, suggesting the hepatoprotective nature of CIGB-258. The results align well with an earlier report, which showed a substantial protective effect of CIGB-258 against liver inflammation [20]. Moreover, the study reported the higher efficacy of CIGB-258 in preventing hepatic inflammation and damage compared with the standard pro-inflammatory cytokine inhibitors, infliximab and tocilizumab [20]. In addition to modulating hepatic inflammatory markers, CIGB-258 markedly suppressed CML-induced ROS production, indicating improved liver health in the CIGB-258-treated group. Among the varied anti-inflammatory events induced by CIGB-258 [15,17], its substantial effect on HDL functionality and stabilization [11,19,20] is also a reason for the higher anti-inflammatory effect in the CIGB-258-injected group, as the immune-modulatory role of HDL has been recognized [57] that regulates inflammatory events and thus has a substantial protective role in sepsis [57,58].

Like the effect on the liver, CIGB-258 protects kidney damage and reduces ROS production and senescence triggered by exposure to CML + Et-OH. Reduced ROS production in response to CIGB-258 treatment is one of the important reasons for lower senescence, as an inverse association between oxidative stress and senescence has been well described [59,60]. In addition, a diminished plasma sulfhydryl content in response to CML + Et-OH was substantially elevated by exposure to CIGB-258, reflecting good kidney health, as the diminished sulfhydryl content is associated with kidney impairment [39].

Severe intestinal bleeding, histological changes, and hypertrophy of the goblet cells were observed in response to exposure to CML + Et-OH, which were substantially prevented by exposure to CIGB-258. Also, it has been described that CML leads to tissue fibrosis by inhibiting PKC activation [61]. Likewise, a notable increase in tissue fibrosis was observed in the CML-injected group, which substantially inhibited exposure to CIGB-258. Among the varied cellular events, severe inflammation and oxidative stress induced by CML have been recognized as key drivers of fibrosis and intestinal damage. The statement is consistent with reports describing the key roles of oxidative stress and inflammation in fibrosis [62,63] and intestinal damage. Consistent with the notion, a high prevalence of ROS around the fibrotic areas was observed in the CML group, which was inhibited by exposure to CIGB-258, suggesting the critical role of oxidative stress in fibrosis and intestinal damage.

The results obtained confirm that the co-treatment with CML + Et-OH in zebrafish constitutes a valid sepsis-like hyperinflammatory model, as it consistently reproduces hallmark events of this condition: rapid mortality, intestinal hemorrhage, dyslipidemia, neutrophil infiltration, elevated IL-6 production, and hepatic and renal damage. These findings are comparable to those described in classical rodent models of sepsis, such as LPS administration or CLP, which are widely used in preclinical research. In particular, the strong induction of IL-6 and neutrophil efflux observed in zebrafish closely mirrors the cytokine dynamics reported in LPS-induced sepsis, where IL-6 and TNF-α are key mediators of hyperinflammation and multi-organ failure. While LPS models often report additional cytokines such as IL-1β and IFN-γ, the zebrafish model captures the core inflammatory signature, reinforcing its translational relevance. The advantage of the zebrafish lies in its high genetic and physiological similarity to humans, its small size, and easy husbandry, which allow efficient evaluation of large cohorts. In this context, the CML + Et-OH model provides a robust platform to explore mechanisms of hyperinflammation and multi-organ failure, as well as to validate novel therapeutic approaches such as CIGB-258.

Limitation of the study: The limited analysis of pro- and anti-inflammatory mediators and the lack of detailed molecular–mechanistic studies elucidating the effects of CIGB-258 on NF-κB modulation and RAGE signaling represent key limitations of the present study that should be addressed in future investigations. Furthermore, the efficacy of CIGB-258 was evaluated using the non-infectious zebrafish model. In future studies, CIGB-258 efficacy should be tested in an infectious sepsis model to validate its therapeutic potential under true septic conditions.

## 4. Materials and Methods

### 4.1. Materials

CIGB-258^®^ (Jusvinza), a synthesized altered peptide (27 amino acids) derived from the heat shock protein HSP60, was provided by the Center of Genetic Engineering and Biotechnology (CIGB), Havana, Cuba, for research purposes only. The CIGB-258 was synthesized on Fmoc-AM-MBHA resin by a stepwise solid-phase procedure using the Fmoc/tBu strategy. The peptide was purified using reversed-phase high-performance liquid chromatography (RP-HPLC), and its identity was confirmed by mass determination using a hybrid orthogonal configuration QTOF-2 mass spectrometer (Waters Micromass, Wilmslow, UK) equipped with a Z-spray electrospray ionization source operating in positive mode (nanoESI+; Waters Micromass, Wilmslow, UK). The CIGB-258 was 98.6% pure and had a molecular weight of 2986.6 Da. *N*-ε-carboxymethyllysine (cat. no. 14580-5g) was procured from Sigma-Aldrich (St. Louis, MO, USA). All the other chemicals and reagents, unless otherwise stated, were of analytical grade and used as supplied.

### 4.2. Zebrafish Rearing

Young zebrafish (AB strain, 18 weeks old) were raised at 28 °C water temperature under alternating light and dark photoperiods of 14 h and 10 h, respectively, following the standard guidelines on Animal Care and Use [64] adopted by the Raydel Research Institute (approval no. RRI-24-001; date of approval: 2 September 2024). The zebrafish were fed twice daily (at 9 am and 6 pm) with a standard commercial fish food (Tetrabit GmbH, D49307, Melle, Germany). The zebrafish were maintained in this environment for 1 week to acclimatize them before the experiment.

The water used for maintaining the zebrafish was strictly monitored for pH, dissolved oxygen (DO), turbidity, chlorine, total bacteria count, and total fecal coliform contamination. Water quality analysis was conducted by Kirim Life Science Co., Ltd. (Daegu, Republic of Korea), which certified that the water quality was safe for human and animal use (Supplementary Figure S4). The supplied water had a pH of 7.3, a turbidity of 1.6 NTU, a residual chlorine content of 1.8 mg/L, and a DO content of 8 mg/L. The general bacterial count was ≤100 CFU, with no detectable coliforms. Furthermore, the water was filtered sequentially through a 5 μm microdepth filter, activated carbon, and a 1 μm microdepth filter, and it was treated with UV light prior to being supplied to the zebrafish. 

### 4.3. Induction of Acute Inflammation and Body Weight Anlysis

**Figure 9:**
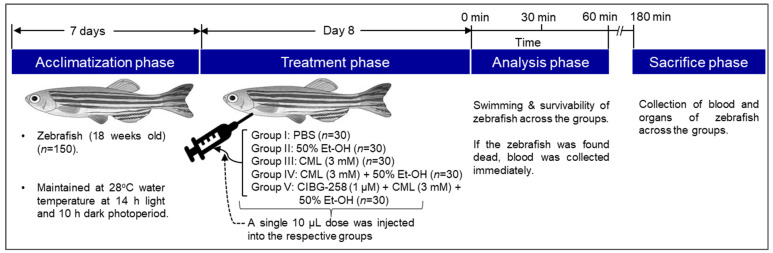
Schematic representation of the experimental plan. Abbreviations: PBS: phosphate-buffered saline; Et-OH: ethanol; CML: carboxymethyllysine; CIGB-258: peptide.

The zebrafish (n = 150) were randomly divided into five different groups (Figure 9). Each group contained 30 fish that were segregated into 3 tanks, each containing 10 zebrafish (10 × 3 tanks = 30). Each zebrafish in group I was intraperitoneally injected with 10 μL of phosphate-buffered saline (PBS, pH 7; control), and the zebrafish in groups II and III were injected with 10 μL of 50% ethanol in PBS (Et-OH, pH 6) and carboxymethyllysine (CML, final 3 mM) in PBS, respectively. The zebrafish in group IV received a 10 μL injection of CML (final 3 mM, pH 7) dissolved in 50% Et-OH, while the zebrafish in group V were injected with 10 μL of CIGB-258 (final 1 μM, pH 6) containing CML (final 3 mM) + 50% Et-OH. Notably, single 10 μL doses of PBS (group I), Et-OH (group II), CML (group III), CML + Et-OH (group IV) and a blend of CIGB-258 + CML + Et-OH (group V) were injected into the respective groups (as mentioned in Figure 9). The CIGB-258 concentration (final 1 μM) was selected based on previous studies documenting that this dose produces a substantial beneficial effect in zebrafish [20,43]. A 28-gauge syringe was used for the intraperitoneal injection after anesthetizing the zebrafish by submersion in a 0.1% 2-phenoxyethanol solution. The 50% Et-OH concentration was selected based on prior screening experiments, where 10 μL of 1–100% Et-OH alone and in combination with CML (3 mM) was examined. The outcomes showed that 50% Et-OH in combination with CML displayed persistent acute death and intestinal bleeding. Beyond the 50% Et-OH concentration, zebrafish abdominal bleeding and mortality remain almost constant. Therefore, 50% Et-OH, as the lowest concentration in conjunction with CML, was used to induce acute toxicity in zebrafish.

The body weights of the zebrafish across all groups were examined prior to the treatment and at 30, 60, and 180 min post-treatment using an electronic weighing machine (Ohaus, Parsippany-Troy Hills, NJ, USA).

The zebrafish were monitored throughout the experiment to detect any instances of aggression or physical injury.

### 4.4. Survivability and Swimming Analysis

Survivability and swimming ability across the groups were assessed at 5 min, 30 min and 60 min post-treatment. Swimming ability was measured by the movement of the tail fin and paucity of body paroxysms [65], while death was assessed by closely examining gill movement, stationary position, head up or down, floating on the water surface or sinking to the bottom, following the Organization of Economic Co-operation and Development (OECD) 2019 guidelines [66].

### 4.5. Quantification of Abdominal Bleeding and Collection of Blood and Organs

After 180 min post-treatment, the injected sites (abdominal region) were visualized, and images were captured using a digital camera (Canon EOS 90D; Tokyo, Japan). The captured images were processed to quantify the bleeding areas (severe abdominal redness) using ImageJ (https://imagej.net/ij, version 1.53; accessed on 6 June 2025).

The zebrafish were sacrificed 180 min post-treatment by hypothermic shock [67], and blood was collected from the heart using a 22-G needle. Blood was collected separately from the zebrafish maintained in the three different tanks (n = 3) for each experimental group (as described in Section 4.3). For each tank within a given group, the collected blood was pooled into a single tube and mixed with ethylenediaminetetraacetic acid (EDTA, 1 mM) at a 2:3 (*v*/*v*) ratio. All collected blood samples were centrifuged at 6000 rpm for 10 min. The supernatant (plasma) was collected and kept in a refrigerator (4 °C) for further use.

Different organs (livers, kidneys and intestines) were surgically removed under a stereomicroscope (Motic SMZ 168; Hong Kong, China) and kept in 10% formalin for further histological analysis.

### 4.6. Oxidative and Antioxidant Parameters, Hepatic Function Biomarkers, and Lipoprotein Profile of Plasma

Plasma malondialdehyde (MDA) level, sulfhydryl content, ferric ion reduction ability (FRA) and paraoxonase (PON) activity were quantified using the earlier described method [12]. A detailed methodology is provided in Supplementary Section S1.

The plasma lipoprotein profiles [total cholesterol (TC), triglycerides (TGs), high-density lipoprotein cholesterol (HDL-C) and hepatic function biomarkers (AST and ALT)] were quantified using commercial kits following the instructions of the manufacturers. A detailed methodology is provided in Supplementary Section S2.

### 4.7. Histological and Immunohistochemical (IHC) Staining

For histological analysis, different tissues (livers, kidneys, and intestines) were individually embedded in Surgipath FSC22 frozen section solution (3001480, lot no. 072325; Leica, Nussloch, Germany). The sample tissue was placed in the center of the object holder, and the FSC22 frozen-section solution was then added to completely cover the tissue. The object holder (with the embedded sample) was then placed in a liquid nitrogen chamber to allow the solution to solidify (~5 min). The solid block containing the tissue was then stored in a deep freezer (–21 °C) for 24 h to stabilize the block. Finally, the solidified tissue block was sectioned (7 μm thick slices) using a cryo-microtome (Leica CM-1510S; Nussloch, Germany).

Morphological changes in the livers, kidneys and intestines were assessed by hematoxylin and eosin (H&E) staining [68]. In brief, each tissue section (7 μm thick) was covered with Ventana HE 600 hematoxylin solution (lot no. N 11615; Roche, Tucson, Arizona, USA) for 5 min, followed by a 1 min water wash and subsequent addition of 0.5% HCl. The section was washed with water (~20 s), followed by the addition of 0.05% ammonia water (~10 s). The section was thoroughly washed with water and stained with Ventana HE 600 eosin solution (lot no. H30444; Roche, Tucson, Arizona, USA). After 1 min, the staining section was washed with ethanol. Finally, the air-dried section was visualized under a microscope (Nikon, Tokyo, Japan).

For the IHC staining, each liver section (7 μm thick) was covered with 200× diluted anti-IL-6 monoclonal antibodies (mouse IgG, ab9324; Abcam, Cambridge, UK). After 16 h of incubation in a cool, moist environment, the section was developed using the EnVision HRP-polymer kit (Dako, Glostrup, Denmark), which contains a horseradish peroxidase (enzyme)-linked secondary antibody (anti-mouse IgG, K4001; Dako, Glostrup, Denmark) and a chromogenic substrate.

### 4.8. Dihydroethidium (DHE), Cellular Senescence and Masson’s Trichome Staining

For the DHE fluorescent staining, tissue sections (7 μm thick) from livers and kidneys were covered with 0.25 mL of DHE solution (final 30 μM). After 30 min of incubation at room temperature (RT) in the dark, each section was thoroughly washed three times with PBS and then examined under a fluorescent microscope (Nikon Eclipse TE2000; Tokyo, Japan) at 585 nm excitation and 615 nm emission.

For cellular senescence, tissue sections of livers and kidneys were covered with 0.75 mL of 5-bromo-4-choloro-3-indolyl-β-D-galactopyranoside solution (X-Gal, 0.1%). After 16 h incubation in a moist environment at RT, the sections were washed and visualized under the microscope to detect the blue-stained senescent positive cells.

To visualize collagenation in the bleeding site, a paraffin section of intestinal tissue was stained using Masson’s trichrome staining following the earlier described method [69] with slight modifications to the staining time. In brief, intestinal tissue fibrosis was detected by Masson-trichrome staining. Each intestinal section (7 μm thick) was immersed in Weigert’s iron hematoxylin solution [prepared by mixing equal proportions of solution A (4 g hematoxylin in 200 mL of 80% ethanol) and solution B (8 g FeCl_3_ in 190 mL distilled H_2_O and 2 mL of HCl)]. After 5 min staining in the dark, the section was washed three times with distilled H_2_O and subsequently immersed in Biebrich scarlet acid fuchsin solution (prepared by dissolving 2.25 g Biebrich scarlet and 0.25 g acid fuchsin in 250 mL distilled H_2_O containing 2 mL glacial acetic acid). After 5 min, the section was washed 3 times with distilled H_2_O and subsequently immersed in 1% phosphomolybdic acid. Following 2 min incubation, the section was treated for 5 min in 1.8% aniline blue solution (prepared by dissolving 4.5 g of aniline blue in 250 mL distilled H_2_O containing 4.5 mL of glacial acetic acid). The section was rinsed with distilled H_2_O, followed by 30 sec exposure to a 1% acetic acid solution. Finally, the section was rinsed twice with distilled H_2_O and visualized under a microscope (Nikon, Tokyo, Japan).

### 4.9. Statistical Analysis

One-way analysis of variance (ANOVA) of the normally distributed data was performed using the SPSS software (version 29; Chicago, IL, USA) at the 95% confidence level. Tukey’s post hoc analysis was conducted with statistical significance (*p* < 0.05) to determine differences between groups. The normal distribution of the data was assessed prior to performing one-way ANOVA. To establish the pairwise statistical differences between the groups, *t*-tests were performed.

## 5. Conclusions

CIGB-258 protects zebrafish from CML + Et-OH-induced sepsis-like hyperinflammation and acute death. A substantial effect of CIGB-258 was noticed to minimize abdominal bleeding, oxidative stress, and impairment of antioxidant variables and lipoprotein profiles altered by CML + Et-OH. CIGB-258 displayed a counter-inflammatory effect, attenuating IL-6 production and neutrophil infiltration, thereby protecting the liver and kidneys from CML + Et-OH-induced damage. CIGB-258 inhibited intestinal bleeding, fibrosis, and ROS generation and mitigated CML + Et-OH-triggered severe intestinal toxicity. In conclusion, CIGB-258 effectively counters CML + Et-OH-induced sepsis-like events and protects zebrafish from acute death and multiple-organ failure. These findings suggest that CIGB-258 may have therapeutic potential for managing systemic inflammation and organ injury in human sepsis. However, additional preclinical and clinical studies are needed to confirm CIGB-258 safety and efficacy in humans.

## Data Availability

The original contributions presented in this study are included in the article/Supplementary Material. Further inquiries can be directed to the corresponding author.

## Supplementary Materials

The following supporting information can be downloaded at https://www.mdpi.com/article/10.3390/ph19030510/s1: Supplementary Videos S1–S6: Swimming ability of zebrafish at 60 min post-treatment. Supplementary Figure S1: Survivability and swimming ability of zebrafish following different treatments. Supplementary Figure S2: Intraperitoneal injection sites and bleeding areas of the zebrafish after the specified treatment. Supplementary Figure S3: A comparative analysis of oxidative variables, antioxidant parameters, and liver function biomarkers of the plasma from the zebrafish that received the specified treatment. Supplementary Figure S4: Certificate of water quality analysis. Supplementary Material Section S1: Methodology for the detection of plasma oxidative and antioxidant variables. Supplementary Material Section S2: Methodology for the quantification of plasma lipid profile and hepatic function biomarkers.
