# Supplementary material for: CIGB-258, a Potential Novel Approach to Treat Sepsis-like Hyperinflammation, Reduces Gastrointestinal Hemorrhage in Zebrafish Exposed to Carboxymethyllysine and Ethanol
Source: Pharmaceuticals (Basel). 2026 Mar 20;19(3):510. doi: 10.3390/ph19030510 (PMC13029758; doi:10.3390/ph19030510)
Supplement: Supplementary file 1 [file pharmaceuticals-19-00510-s001.zip › pharmaceuticals-4161457-supplementary/Supplementary Material-pharmaceuticals.pdf]

Supplementary Material

Supplementary Figure S1

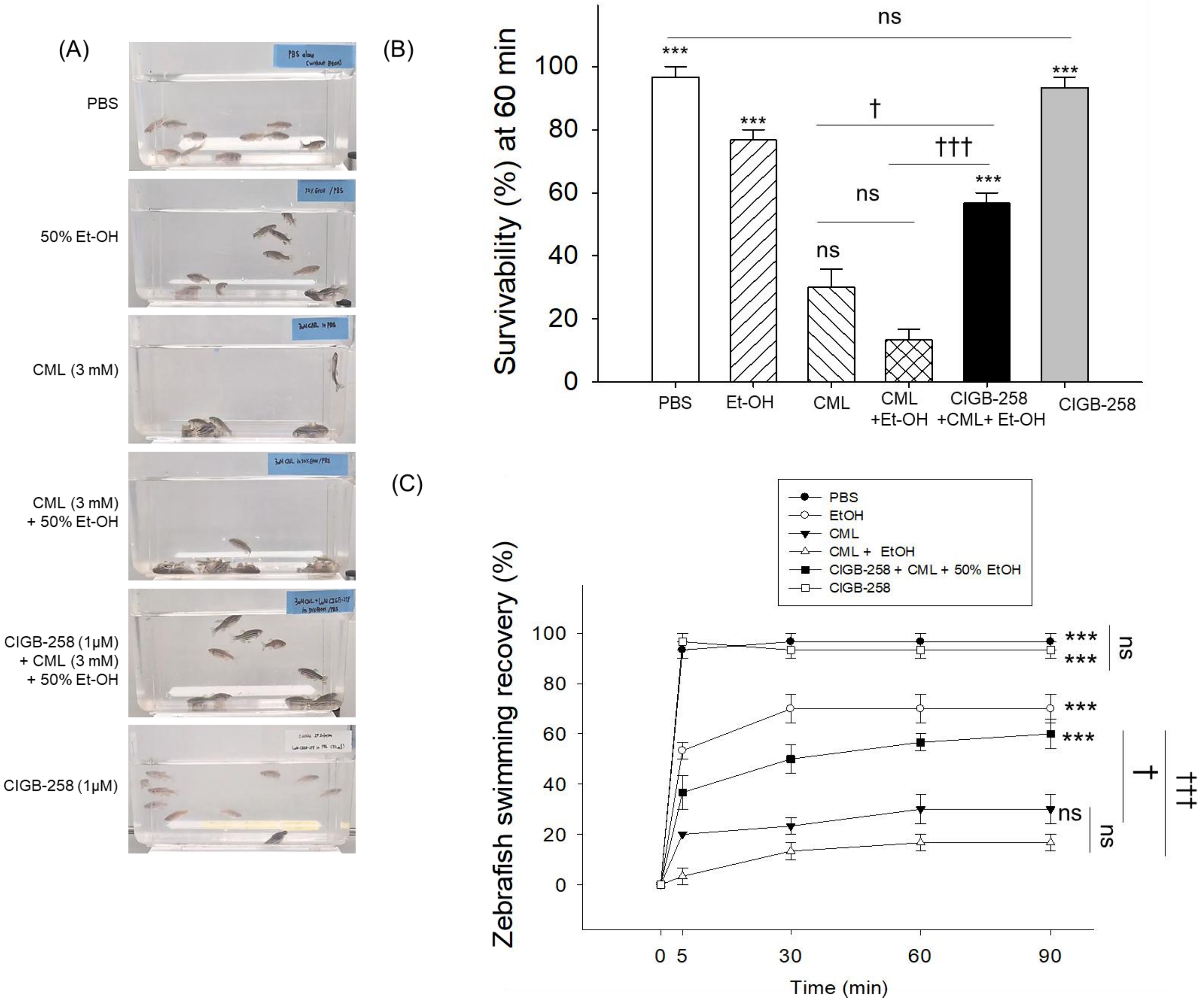

Supplementary Figure S1: Survivability and swimming ability of zebrafish following the intraperitoneal injection of ethanol (Et-OH), carboxymethyllysine (CML), individually and in combination with CIGB-258. (A) Snapshot of the swimming pattern at 60 min post-injection. Text shown in the blue boxes is positioned on the left side of the panel A. (B) Survivability, and (C) kinetics of swimming recovery during 60 min post-injection. The *** (p<0.001) highlights the statistical difference compared to the CML + Et-OH using one way ANOVA following Tukey's post hoc analysis. The † (p<0.05) and ††† (p<0.001) highlights the pairwise statistical difference between the marked groups using *t*-test; the "ns" represent the non-significant difference.

Supplementary Figure S2

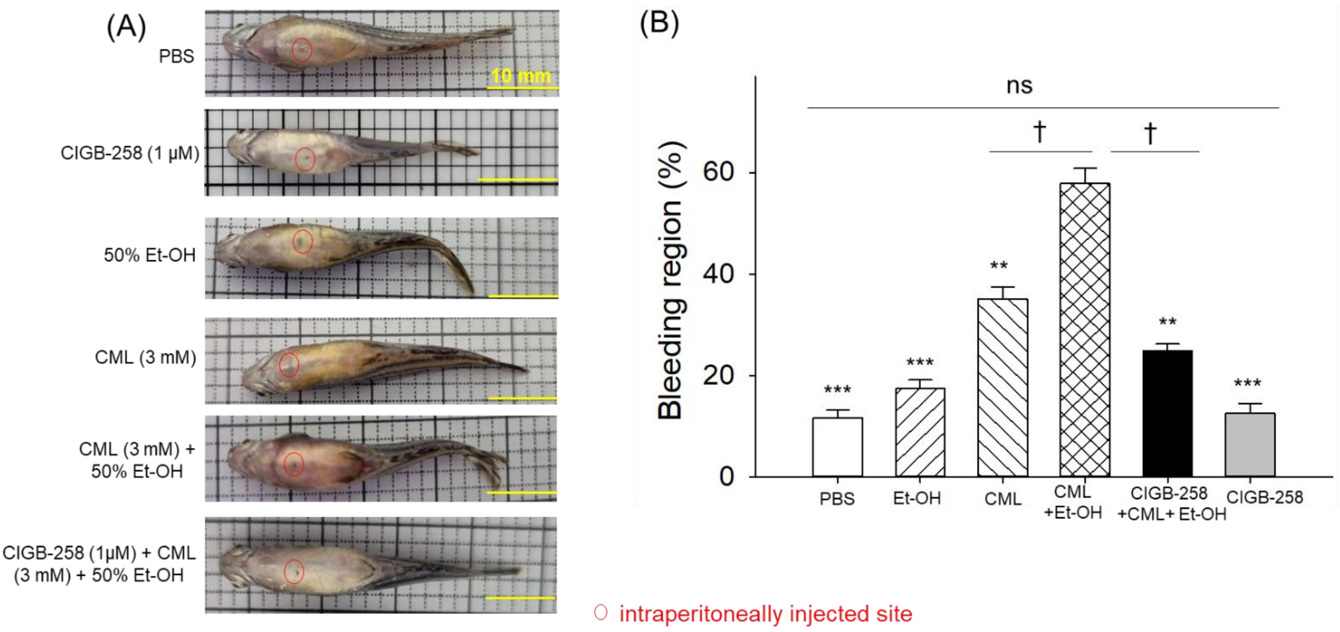

Supplementary Figure S2: Intraperitoneal injection sites and bleeding areas of the zebrafish from different groups received the specified treatment. (A) A representative image of the zebrafish abdominal injection site, and (B) quantification of the abdominal bleeding area after 180 min treatment. Abbreviations: PBS: phosphate-buffered saline; Et-OH: ethanol; CML: carboxymethyllysine, and CIGB-258: peptide. Data points in the bar graph represent a mean ± SEM (n=30). The ** (*p*<0.01) and *** (*p*<0.001) highlight the statistical difference compared to the CML+Et-OH using one-way ANOVA following Tukey's post hoc analysis; the † (*p*<0.05) highlights the statistical difference between the marked group using a *t*-test; the "ns" represent the non-significant difference.

.

Supplementary Figure S3

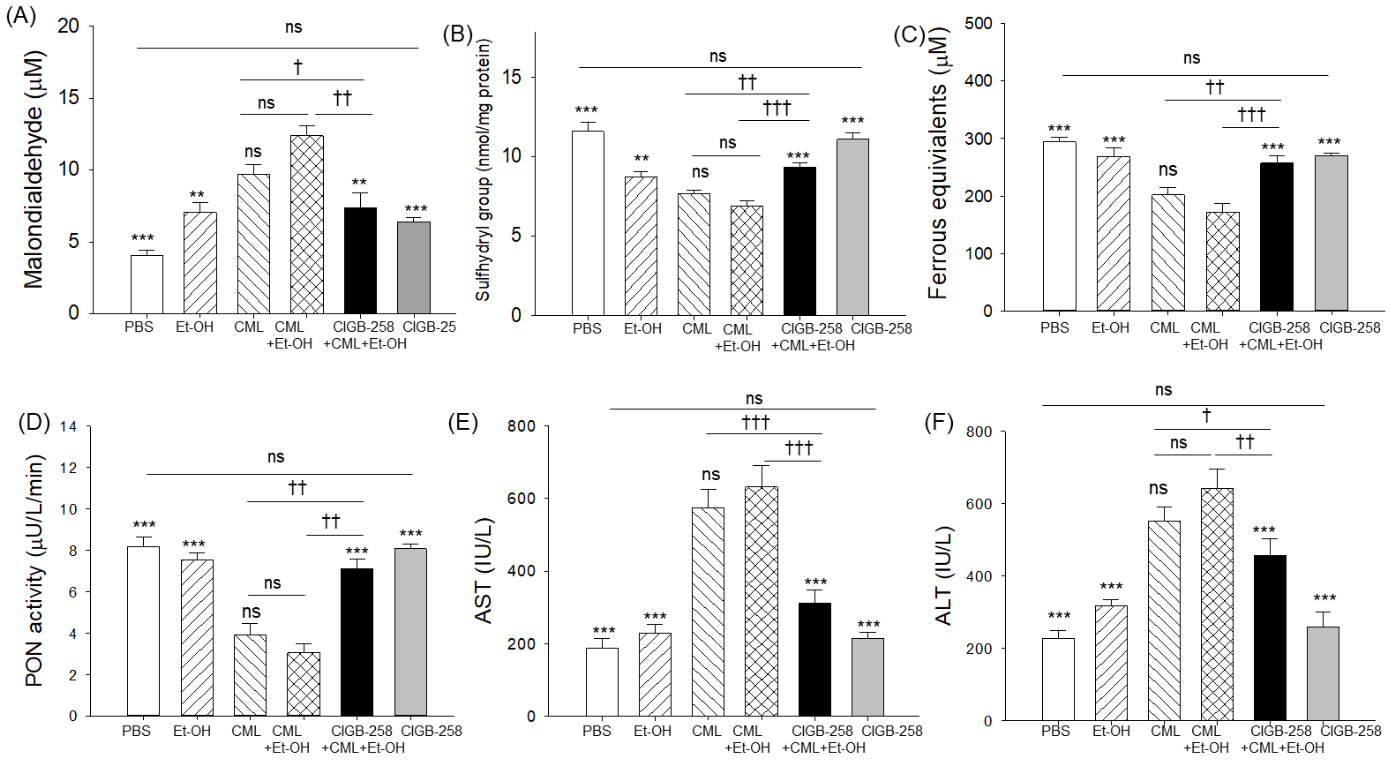

Supplementary Figure S3: A comparative analysis of oxidative variables, antioxidant parameters, and liver function biomarkers of the plasma from the zebrafish that received the specified treatment. Plasma (A) malondialdehyde, (B) sulfhydryl, (C) ferrous equivalent, (D) paraoxonase (PON), (E) aspartate aminotransferase (AST), and (F) alanine aminotransferase (ALT) levels. Abbreviations: PBS: phosphate-buffered saline; Et-OH: ethanol; CML: carboxymethyllysine, and CIGB-258: peptide. Data points in the bar graph represents a mean ± SEM (n=3). The ** (p<0.01) and *** (p<0.001) highlight the statistical difference compared to the CML + Et-OH using one way ANOVA following Tukey's post hoc analysis; the † (p<0.05), †† (p<0.01) and ††† (p<0.001) highlights the statistical difference between the marked group using t-test; "ns" represent the non-significant difference.

Supplementary Figure S4

Certificate of water quality

Drinking Water Quality Test Report
Recipient: Korea Mediventure Center
B2 Management Office
76 Dongnae-ro, Dong-gu, Daegu (Dongnae-dong), 41061
Issued by: Kirim Life Science Co., Ltd.
Issue Date: May 2, 2025
Report Number: KWCT3379961
Subject: Drinking Water (Reservoir Water) Quality Test Report

Basis of Issuance
This report is issued in accordance with Article 3, Paragraph 2 of the *Regulations on Drinking Water Quality Standards and Testing*.
It presents the results of the sample provided by the client. The report must not be used for purposes other than water quality testing.

1. Sample Information
•**Receipt Number:** 12506438
•**Receipt Date:** April 28, 2025
•**Client:** Korea Mediventure Center
•**Sample Name:** Reservoir Water
•**Purpose of Test:** Waterworks Act (Reservoir Water Quality Test)
•**Sampling Location:** 76 Dongnae-ro, Dong-gu, Daegu (Dongnae-dong)
•**Collected by:** Technical staff (Seong Ki-hyun, Kirim Life Science Co., Ltd.)
•**Sampling Date/Time:** April 28, 2025
•**Remarks:** Evaluation conducted in accordance with Article 22, Paragraph 4 of the Enforcement Rules of the Waterworks Act and Annex 1 of the *Regulations on Drinking Water Quality Standards and Testing*.

2. Test Results

Test Item	Standard	Result
Turbidity	≤ 5 NTU	1.6
pH (Hydrogen Ion)	5.8 – 8.5	7.3
Residual Chlorine	0.1 – 4.0 mg/L	1.8
General Bacteria	≤ 100 CFU/mL	0
Total Coliform Group	Not detected / 100 mL	Not detected
Fecal Coliform Group	Not detected / 100 mL	Not detected

Comprehensive Result: Suitable (Compliant)

Note: Turbidity (non-graded performance) and residual chlorine (Grade 1) were measured using a simplified measuring device.

Certification
Issued by: **Kirim Life Science Co., Ltd.** Representative Director
- A beautiful company that values environment and life -

Supplementary Figure S4: Certificate of water quality analysis.

Section S1.

1. Malondialdehyde (MDA), sulfhydryl group, ferric ion reduction (FRA) activity and paraoxonase (PON) activity

The blood malondialdehyde (MDA) level was quantified by mixing plasma sample (20 μL, equivalent to 1 mg/mL protein) with trichloroacetic acid (50 μL, 0.2 mg/μL, pH 1.4) and thiobarbituric acid (100 μL, 6.7 μg/μL, pH 2.3). Following a 10-min incubation at 95 °C, the absorbance at 560 nm was recorded.

The sulfhydryl group was quantified by mixing 60 μL of plasma (1 mg/mL protein) with 60 μL of 5,5′-dithio-bis-(2-nitrobenzoic acid) (DTNB) (4 mg/mL). After 12 hr incubation at room temperature, absorbance 412 nm was determined, and sulfhydryl groups were quantified utilizing 13,600 M^{-1} cm^{-1} extinction coefficient (ε) of DTNB.

To assess ferric ion reduction (FRA) capacity, 20 μL of the plasma (1 mg/mL equivalent protein) was mixed with 180 μL of FRA reagent (prepared by blending 10 mL of acetate buffer (0.2M, pH 3.6) with 1.25 mL each of 2,4,6-tripridyl-S triazin (10 mM, pH 1.9) and $FeCl_3$ (20 mM, pH 2.1). After incubating the mixture at RT for 60 min, absorbance was measured at 593 nm. The results were quantified in μM ferric equivalents based on a ferrous sulfate standard curve.

For paraoxonase activity 20 μL of plasma (1 mg/mL equivalent protein) was mixed with 180 μL of buffer (pH 8.3) [Tris-HCl (90 mM), NaCl (3.6 mM), $CaCl_2$ (90 mM)] containing the paraoxon-ethyl substrate (0.55 M). After 60 min incubation at 25°C, an absorbance (415 nm) was recorded using Microplate reader (Model, iMark™ S.N 21275, Bio-Rad, Hercules, CA, USA) to quantify the production of *p*-nitrophenol, a hydrolysis product of paraoxon-ethyl. Results are expressed as μU/L/min employing the extinction coefficient 17,000 $M^{-1}cm^{-1}$ for *p*-nitrophenol.

Section S2.

2. Method to quantify plasma levels of total cholesterol (TC), triglycerides (TGs), high-density lipoprotein cholesterol (HDL-C) and hepatic function biomarkers aspartate transaminase (AST) and alanine transaminase (ALT)

The plasma total cholesterol (TC) and triglycerides (TGs) were determined using commercial assay kits (cholesterol, AM 202-K, and TGs, AM 157-K, Asan Pharmaceutical, Hwasung, Republic of Korea) as per the method suggested by the suppliers. In brief, 5 μL serum was mixed with 200 μL reaction mixture (supplied with a commercial assay kit) for the TC analysis. The content was incubated at 37°C for 10 min, resulting in a red-colored product quantified by adsorption at 490 nm (Microplate reader, iMark™ Bio-Rad, Hercules, CA, USA).

Similarly, 5 μL serum was mixed with a 200 μL of TGs-specific reaction mixture (supplied with a commercial assay kit) for TGs analysis. The content was incubated for 10 min at 37°C, and the formed colored product was quantified by taking adsorption at 490 nm.

For HDL-C analysis, serum was mixed in an equal ratio with the separation solution (supplied with a commercial assay kit), followed by centrifugation at 3,000 rpm for 10 min at 25°C. The supernatant (20 μL) was collected and blended with a 200 μL reaction mixture (supplied with a commercial assay kit). After 10 min incubation at 37°C, red color intensity corresponding to HDL-C was quantified by taking absorption at 490 nm (Microplate reader, iMark™ Bio-Rad, Hercules, CA, USA).

The commercial diagnostic kit (AM102K and AM103-K, Asan Pharmaceutical, Hwasung, Republic of Korea) was used to quantify aspartate transaminase (AST) and alanine transaminase (ALT) levels in the plasma, following the instructions suggested by the manufacturers. Briefly, 5 μL of plasma was combined with 250 μL of either AST or ALT-specific solution, as supplied in the diagnostic kit. Following a 30 min incubation for AST or 60 min incubation of ALT at 37°C, the mixture was then blended with 250 μL of the respective coloring reagent (AST or ATL-specific, provided in the diagnostic

kit). After a subsequent 20 min incubation at room temperature, 250 μL of 0.4 N NaOH was introduced to halt the reaction. Finally, the AST and ATL were quantified by measuring absorbance at 490 nm (Microplate reader, iMark™, Bio-Rad, Hercules, CA, USA).
